# Chronic Aerobic Training at Different Volumes in the Modulation of Macrophage Function and *in vivo* Infection of BALB/c Mice by *Leishmania major*

**DOI:** 10.3389/fmicb.2021.734355

**Published:** 2021-09-20

**Authors:** T. T. Guimarães, S. M. R. Gomes, R. A. A. C. Albuquerque, A. K. C. Lima, G. F. Braga, J. B. Souza, M. Assis, A. C. S. Brito, R. F. Santos, T. Da Silva, L. M. Siqueira, B. D. Ventura, L. S. Rodrigues, R. Terra, S. A. G. Da Silva, P. M. L. Dutra

**Affiliations:** ^1^Discipline of Parasitology, Department of Microbiology, Immunology and Parasitology, State University of Rio de Janeiro, Rio de Janeiro, Brazil; ^2^Laboratory of Immunopathology, Faculty of Medical Sciences, State University of Rio de Janeiro, Rio de Janeiro, Brazil; ^3^Colégio Brigadeiro Newton Braga (CBNB), Diretoria de Ensino (DIRENS), Força Aérea Brasileira (FAB), Rio de Janeiro, Brazil

**Keywords:** exercise, exhaustion, overtraining, *Leishmania* infection, immune response, cellular immunity

## Abstract

Physical inactivity is one of the main causes of chronic diseases; however, strenuous exercise can induce immunosuppression. Several studies suggest that moderate amounts of exercise lead to a Th1 response, favoring the resolution of infections caused by intracellular microorganisms, while high volumes of exercise tend to direct the response to Th2, favoring infection by them. Leishmaniasis is a parasitic disease promoted by parasites of the *Leishmania* genus, with clinical manifestations that vary according to the species of the parasite and the immune response of the host. The experimental *Leishmania major*–BALB/C mouse model provides a good model for the resistance (Th1 response) or susceptibility (Th2 response) that determines the progression of this infection. The aim of this study was to evaluate the effect of aerobic training at different volumes on modulation of *in vitro* macrophage infection by *L. major*, as well as to assess the effect of high volume (HV) aerobic training on the development of *L. major in vivo* in BALB/c mice. Uninfected animals were submitted to various exercise volumes: none (SED), light (LV), moderate (MV), high (HV), very high (VHV), and tapering (TAP). The macrophages of these animals were infected by *L. major* and the LV and MV groups showed a decrease in the infection factor, while the VHV showed an increase in the infection factor, when treated with LPS. The cytokine concentration pattern measured in the supernatants of these macrophages suggested a predominant Th1 response profile in the LV and MV groups, while the Th2 profile predominated in the VHV and TAP groups. Groups of BALB/C mice infected with *L. major* were subjected to high volume (iHV) or non-periodized high volume (iNPHV) exercise or kept sedentary (iSED). The exercised animals suffered a significant increase in injuries caused by the parasites. The animals in the group submitted to high volume exercise (iHV) showed visceralization of the infection. These data strongly suggest that a very high volume of aerobic training increased the susceptibility of BALB/C mice to *L. major* infection, while moderate distribution of training loads promoted immunological balance, better controlling the infection by this parasite.

## Introduction

The engagement in regular and properly oriented physical exercise programs is recognized as a determinant for the promotion and maintenance of health and quality of life (Fiuza-Luces et al., 2013; Heinonen et al., 2014). In recent years, studies have shown that a moderately active lifestyle is associated with health promotion and disease prevention (Hallal et al., 2012; Fiuza-Luces et al., 2013).

Although the average life expectancy in the world has increased, gradually, more people are affected by chronic non-communicable diseases, such as cardiovascular diseases; diabetes; various types of cancer; and mental, bone, and joint disorders (Handschin and Spiegelman, 2008; Lee et al., 2012). According to the World Health Organization (2014), in addition to suffering, functional dependence, intangible expenses in health systems, and reduced quality of life, these diseases are responsible for 58.5% of all deaths in the world. Physical inactivity appears as one of the main causes attributed to mortality (Hallal et al., 2012). On the other hand, people who exercise excessively have the same death risk as people who are not physically active (Arem et al., 2015). Exercise seems to produce health benefits in moderate doses (Arem et al., 2015). Although the intense effort can improve performance and health (Gohil and Brooks, 2012; Sobral-Monteiro-Junior et al., 2019), strenuous loads of physical and mental stress can compromise health. Amateur, professional, or recreational athletes are often affected by metabolic, immunological, neurological, endocrine, cardiovascular, muscular, and skeletal disorders in a process called overtraining (Meeusen et al., 2013; Guimarães et al., 2017).

Overtraining is the condition of poor adaptation to a chronic period of excessive stress promoted by physical effort, which results in loss of performance and development of this syndrome (Kreher and Schwartz, 2012). The signs and symptoms that characterize the overtraining syndrome are mood and anxiety disorders, depression, general apathy, emotional instability, loss of appetite, sleep disturbance, hormonal changes, increased resting heart rate, and increased vulnerability to infections and injuries, in addition to muscle and joint pain (Kellmann, 2010; Reardon and Factor, 2010; Schaal et al., 2011; Portugual et al., 2013). There is a classic hypothesis that overtraining originates when new vigorous exercise sessions are performed without the necessary time to recover from immunosuppression (Smith, 2000; Nielsen and Pedersen, 2008). Exhaustive exercise can lead to acute transient leukocytosis followed by partial suppression of cellular immunity, characterized by a reduction in the number or function of leukocytes and other components of the immune system (Silva and Macedo, 2011; Terra et al., 2012b). The period in which agents of the immune system are suppressed after exhaustion caused by a training session or competitive event is known as a window of opportunity (Febbraio and Pedersen, 2005). The increased risk of upper respiratory tract infections or other deleterious conditions of the opportunistic pathogens can vary within 1–9 h (Pedersen and Fischer, 2007), 72 h (Steensberg et al., 2000), or even 2 weeks (Fischer et al., 2004). Immune imbalance seems to be the origin of the syndrome of overtraining (Smith, 2000; Nielsen and Pedersen, 2008).

While moderate-intensity exercise promotes protection against infections caused by intracellular microorganisms, as it directs the immune response to the predominance of a Th1-type response profile (Terra et al., 2012a, 2019), vigorous activities generate increasing concentrations of anti-inflammatory cytokines (Farhangimaleki et al., 2009; Ru and Peijie, 2009; Zhao et al., 2012; Gholamnezhad et al., 2014). This condition induces the predominance of a Th2 response profile to repair muscle tissue damage and systemic inflammation, although this can result in increased susceptibility to infections by intracellular microorganisms (Farhangimaleki et al., 2009; Ru and Peijie, 2009; Zhao et al., 2012; Gholamnezhad et al., 2014).

The effect of physical exercise on the modulation of the immune system has also been studied through the experimental model with BALB/c mice. Different strains of mice have different susceptibility and resistance to infection by parasites. An excellent model of study on this susceptibility/resistance dichotomy is the infection of BALB/c (susceptible) and C57BL/6 (resistant) mice by the macrophage intracellular protozoan *Leishmania major*. The resistance of C57BL/6 mice is related to the Th1 response leading to macrophage classical activation and parasite destruction (Fritzsche et al., 2010), resulting in self-limited and non-progressive infection. On the other hand, BALB/c mice are susceptible to the progressive development of non-healing injuries, which is associated with the Th2-type response and alternative activation of macrophages (Mosmann et al., 1986; Barthelmann et al., 2012).

Intracellular protozoans of the genus *Leishmania* are the etiological agents of leishmaniasis, a group of parasitic diseases endemic in 102 countries and territories spread across the Americas, Africa, Asia, and Europe (Alvar et al., 2012; World Health Organization, 2021). With a prevalence of 12 million infected people and with about 350 million individuals at risk of infection worldwide, approximately between 700,000 and 1 million cases occur each year (World Health Organization, 2021). Sand fly insects are the transmitters of this parasitosis, where the macrophage is the main host cell of the parasite (Lainson et al., 1977). Depending on the species of parasite in question and the immune response of the host, clinical manifestations range from simple tegumentary involvement (cutaneous leishmaniasis), through more severe mucosal infections (mucosal leishmaniasis), to severe visceral infection (visceral leishmaniasis), which can lead to death (Stark et al., 2006; Shah et al., 2010; Badirzadeh et al., 2013; McCall et al., 2013; Pan American Health Organization, 2021). Cutaneous leishmaniasis is caused by several dermotropic *Leishmania* species, such as *L. braziliensis* and *L. amazonensis* in the New World and *L. tropica* and *L. major* in the Old World (Carvalho et al., 2005; Samy et al., 2014; Kahime et al., 2016). Infection by *L. major* usually produces a single lesion restricted to the site of the sand fly bite (Sacks and Noben-Trauth, 2002).

BALB/c mice infected with *L. major* provide a suitable model for studies of modulation of the immune response in leishmaniasis. When infected with *L. major*, BALB/c mice typically present a Th2 response, and they do not control the infection and progress to progressive skin lesion and visceralization of the parasite (Scott et al., 1989; Sacks and Noben-Trauth, 2002).

In a previous study, our group found that BALB/c mice submitted to moderate-volume exercise did not develop lesions after *L. major in vivo* infection, in addition to presenting a cytokine production profile closer to the Th1 pattern (Terra et al., 2019). Thus, the aim of the present study was to evaluate the effect of aerobic training at different volumes on modulation of *in vitro* macrophage infection by *L. major*, as well as to assess the effect of high volume (iHV) or non-periodized high volume (iNPHV) aerobic training on the development of *L. major in vivo* in BALB/c mice and to assess its production of cytokines.

## Materials and Methods

### Chemicals

Kits for measuring cytokines were purchased from BD Biosciences-US (Becton, Dickinson and Company, NJ, United States). Fetal calf serum (FCS) was purchased from Cultilab Co. (Campinas, São Paulo, Brazil). DMEM culture medium, Schneider’s medium, bacterial lipopolysaccharide (LPS), concanavalin A (ConA), and all other chemicals used in this study were purchased from Sigma (St. Louis, MO, United States). Ketamine and xylazine from Syntec do Brasil LTDA.

### Animals

Male BALB/c mice (*Mus musculus*), 6 weeks old (weight ≈ 30.3 g), were kept in mini-isolators at 22–24°C on shelves ventilated by an IVC filter system (Model Domi AL20 system; Alesco^®^) with a 12-h light/dark cycle. Food and water were provided *ad libitum*. Animals were euthanized using ketamine hydrochloride (7.5 mg), 48 h after the last training session. Animals were divided into five cohorts established based on the results of the first capacity test: sedentary control (SED, *n* = 6), low volume (LV, *n* = 6), medium volume (MV, *n* = 7), high volume (HV, *n* = 8), and very high volume (VHV, *n* = 10). Three more groups were submitted to infection by *L. major* and to exercise: sedentary control (iSED, *n* = 5), high volume (iHV, *n* = 5), and non-periodized high volume (iNPHV, *n* = 6). This study was approved by the Ethics Committee on Experimental Use and Animal Care of the Instituto de Biologia Roberto Alcântara Gomes (protocol number CEA/043/2015), in accordance with the ethical standards of the IJSM (Harriss and Atkinson, 2011).

### Exercise Protocol and Physical Tests

Animals were subjected to a previously described swimming exercise protocol (Terra et al., 2012a) for 30–90 min, two to five times per week, for 10 weeks for *in vitro* infection (LV, MV, HV, and VHV) or 12 weeks for *in vivo* infection (iHV and iNPHV), according to the exercise volume desired (see Table 1). To reach this desired exercise volume, weight also was attached to their tails. The initial mass was 2% of the measured body mass (BM) of each individual animal from the MV, HV, VHV, iHV, and iNPHV groups. The load was increased to 3.5% BM in the second week and kept until the 10th or 12th week. Animal BM was measured weekly.

**TABLE 1:** Swimming exercise protocol data of the experimental cohorts.

**Group**	**Time (min)**	**Frequency/week**	**Charge (% BM)**	**Total of weeks**	**Physical test**
SED	–	–	–	–	1st, 6th, and 11th
LV	30	2×	–	10	1st, 6th, and 11th
MV	30	3×	2% (1st week) 3.5% (from 2nd week)	10	1st, 6th, and 11th
HV	60 (until 3rd week) 90 (from 4th week)	3×	2% (1st week) 3.5% (from 2nd week)	10	1st, 6th, and 11th
VHV	60 (until 3rd week) 90 (from 4th week)	5×	2% (1st week) 3.5% (from 2nd week)	10	1st, 6th, and 11th
TAP (5 mice from VHV)	60	2×	–	2	13th
iSED	–	–	–	–	1st, 6th, and 13th
iHV	60 (until 3rd week) 90 (from 4th week)	3×	2% (1st week) 3.5% (from 2nd week)	12	1st, 6th, and 13th
iNPHV	60 (until 3rd week) 90 (from 4th week)	5×	2% (1st week) 3.5% (from 2nd week)	12	1st, 6th, and 13th

*In vitro infection groups: sedentary control (SED), low volume (LV), medium volume (MV), high volume (HV), very high volume (VHV), and tapering (TAP).*

*In vivo infection groups: sedentary control (iSED), high volume (iHV), and non-periodized high volume (iNPHV).*

At the end of the 10-week training period, the sixth experimental non-infected group was created, starting from the VHV group, called tapering (TAP, *n* = 5). Five animals that performed the training protocol of very high volume were submitted to tapering, which consisted of an active rest, with a total duration of 2 weeks. There was a reduction in frequency, duration, and intensity in this period. The frequency was reduced to two weekly sessions, lasting 60 min and without additional overload on the tail.

The animals from the exercised groups were submitted to three physical fitness tests: (1) in the first week before the exercise protocol started, (2) in the sixth week, and (3) in the 11th week for non-infected animals or in the 13th week for the infected one. The TAP group had its physical capacity assessed in the 13th week (fourth physical capacity test). The test consisted of a timed swimming session with a 2% BM load until the point of fatigue, which was defined as the moment when the animal remained submerged for 10 s without returning to the surface for breath.

### Food Consumption and Body Mass Assessment

Food consumption was calculated weekly, on the same day and time, and is represented by the difference, in grams, between the food offered and the residual. Subsequently, the average consumption per animal was calculated. The body mass of the animals was checked once a week, before the training sessions, where a digital scale with 0.1 g precision was used.

### Microorganisms

In this work, virulent promastigotes of *L. major* LV39 (MRHO/Sv/59/P) strain were used, kindly provided by Dr. George dos Reis, Instituto de Biofísica, Universidade Federal do Rio de Janeiro. The virulent strain of *L. major* was maintained through inoculation in BALB/c mice (*M. musculus*). Stationary phase promastigotes (10^7^ parasites) were inoculated in the right footpad. After 2 months of infection, the infected animals were euthanized to remove the inguinal and popliteal lymph nodes and puncture the paw lesion. These tissues were macerated in Schneider’s medium, added with streptomycin/penicillin 0.1%. The result of the maceration was inoculated into 15 ml screw cap tubes, containing 5.0 ml of Schneider’s medium, supplemented with 20% fetal bovine serum and blood agar, and kept at 26°C. After isolation, the parasites were maintained under previously described conditions (Cuba et al., 1986) in Schneider’s medium supplemented with 2 mM glutamine, 100 units/ml penicillin, 100 mg/ml streptomycin, and 10% fetal calf serum in a humidified incubator at 26°C. The parasites were cryopreserved, just once, soon after isolation (P1) and kept in liquid nitrogen until the beginning of this work. Subsequently, they were thawed and expanded *in vitro* to carry out the infection experiments being, at most, in the fourth passage (P4) during the performance of these experiments. After defrosting, the promastigotes were maintained in culture (*in vitro*), for no more than four passages, to maintain infectivity, when again they were inoculated into BALB/c mice.

### Infection Model

Infection of the BALB/c mice was performed through the inoculum of 2 × 10^6^ *L. major* promastigotes (in 20 μl) in the plantar right footpad of these animals. Infected animals were divided into three groups: untrained animals (iSED), animals submitted to high volume exercise (iHV), and animals submitted to very high volume exercise (iNPHV). To monitor the development of the lesion, the infected and contralateral paws were measured weekly with a caliper (Mitutoyo, Brazil). The thickness of the lesion was calculated based on the difference between the thickness of the infected and uninfected (contralateral) paw.

### Antigen Parasite Preparation

The parasites (in the first passage *in vitro*) in the stationary phase (determined by a growth curve) were washed three times and resuspended in DMEM medium, and the parasite number was adjusted to 2 × 10^8^/ml. They were submitted to freezing–thawing cycles, three altogether, in liquid nitrogen for complete lysis. The antigen was stored in aliquots at −20°C until use. The Lowry method (Lowry et al., 1951) was used to quantify the protein concentration in the lysate.

### Delayed Type Hypersensitivity

To evaluate delayed type hypersensitivity (DTH), 20 μg of the total antigen (in 20 μl) was inoculated into the plantar cushion of non-infected paw. After 48 h from the inoculation, the extent of swelling was measured using a caliper rule (Mitutoyo). The DTH was expressed as the difference between the paw thickness before and after the antigen inoculation.

### Euthanasia

The SED, LV, MV, HV, and VHV groups were euthanized about 48 h after the physical capacity test 3. The euthanasia of mice in the TAP group occurred 48 h after the physical capacity test 4. For animals in training with *in vivo* infection (iSED, iHV, and iNPHV), euthanasia was performed 48 h after the last physical capacity test. The procedure in all cases was performed through the injection of ketamine hydrochloride (7.5 mg).

### Obtaining Peritoneal Macrophages and Infection With *L. major*

Macrophages were obtained by peritoneal washing with cold RPMI culture medium, from the SED, LV, MV, HV, VHV, and TAP animal groups. The macrophages were quantified, washed three times (1,500 g, for 10 min at 4°C), plated on glass coverslips in 24-well plates (1 × 10^6^ cells/well), and incubated at 37°C, in a 5% CO_2_ atmosphere for 30 min. After this period, the wells were washed with RPMI medium to remove non-adherent cells, and the plates were incubated in a 5% CO_2_ atmosphere at 37°C for 24 h in RPMI medium in the absence or in the presence of 10 ng/ml bacterial LPS. The *L. major* promastigotes were left in contact with the macrophage monolayers (5:1) in an atmosphere of 5% CO_2_ at 37°C for 2 h, when the supernatant was removed and the cells were washed three times (PBS) to remove non-internalized parasites. Then, the cells were incubated in RPMI medium in an atmosphere of 5% CO_2_ at 37°C for 24 h. After this time, the supernatants were collected and stored at −20°C, for cytokine and nitric oxide (NO) dosages. The coverslips were stained with Giemsa and at least 200 macrophages were counted to determine the infection factor.

Infection factor (IF) = percentage of infected macrophages × the average number of amastigotes per macrophage.

### Nitric Oxide Dosage

NO was evaluated using the Griess method (Green et al., 1982). The macrophage supernatant (100 μl) was plated in a 96-well microplate. The two Griess reagent solutions (0.1% naphthylethylenediamine in 5% phosphoric acid and 1% p-aminobenzene sulfonamine in 5% phosphoric acid) were mixed in a 1:1 ratio at the time of use; 100 μl of the Griess reagent was added to each sample and the systems were incubated for 15 min, in the absence of light, at room temperature. Then, the samples were read in a TP reader Thermo Plate microplate reader at 550 nm. NO was calculated using the standard sodium nitrite curve (10–200 μM) (Stuehr et al., 1991).

### Quantification of the Parasitic Load on the Popliteal Lymph Nodes by Limiting Dilution Assay

After the infected animals were euthanized, popliteal lymph node cells draining infection were aseptically obtained, weighed, macerated, and plated in 96-well microplates in serial dilutions from 1:5 to 1:78,125 in Schneider’s medium supplemented with 20% SFB and streptomycin/penicillin 0.1%. The systems were incubated at 26°C until the growth of parasites was observed in the last dilution of the control system (sedentary—SED), when the parasites were quantified by counting in a Neubauer chamber. The emergence of parasites occurred 24 h after maceration and plating. The result of the counts was adjusted for the concentration of parasites in the macerate and divided by the gram weight of the lymph node.

### Spleen Cell Culture and Infection Visceralization

The spleens from infected animals were macerated using a syringe plunger. The macerate was centrifuged at 1,500 *g* for 10 min at 4°C, and the pellet was resuspended in ACK buffer (0.15 M NH_4_Cl, 1.0 mM KHCO_3_, 0.1 mM Na_2_EDTA, pH 7.2) and incubated for 1 min in an ice bath. After incubation, the ACK was removed by the addition of RPMI (5 ml) and cells were centrifuged at 1,500 *g* for 10 min at 4°C. The cells were resuspended in RPMI medium, quantified in a Neubauer chamber using 0.02% Trypan blue, plated at 1 × 10^6^ cells per well in a 24-well plate, and incubated at 37°C for 48 h in a 5% CO_2_ atmosphere. The supernatants were collected and stored at −80°C for cytokine measurement.

The parasitic load on the spleen was quantified by limiting dilution assay. A small volume of the macerate was plated in 96-well microplates in serial dilutions from 1:5 to 1:3,125 in Schneider’s medium supplemented with 20% SFB and streptomycin/penicillin 0.1%, and the plates were incubated at 26°C until the growth of parasites was observed in the last dilution. The plates were examined daily to check the growth of the parasites. The emergence of parasites occurred 4 days after maceration and plating. The result of the counts was adjusted for the concentration of parasites in the macerate and divided by the gram weight of spleen. The plates were incubated during 14 days to verify the parasite growth in the iSED and iNPHV groups.

### Cytokine Dosage by Flow Cytometry (Cytometric Bead Array)

Cytokine levels of TNF, MCP-1, IL-10, IL-6, IL-2, IFN-γ, IL-17A, and IL-4 in the supernatants from cultured spleen cells and macrophage were measured using the cytometric bead array (CBA) mouse inflammation kit or Th1/Th2/Th17 kit (BD Biosciences, CA, United States), according to the recommendations of the manufacturer. Briefly, the samples were incubated for 3 h at room temperature with a mixture of fluorescent capture spheres covered with specific antibody for each cytokine and fluorescently labeled detection antibodies. After this time, the samples were washed with the solution provided by the manufacturer. Around 2,500 events were acquired in FACS Canto II flow cytometer (Becton Dickinson, Mountain View, CA, United States) using the FACS Diva software. Data analysis was performed using the FCAP Array program (BD Biosciences) and the concentration of cytokines was determined based on the established standard curve, according to the guidelines of the manufacturer.

### Statistical Analysis

Initially, Kolmogorov–Smirnov analysis was performed to test the normality of the data. Given this assumption, the data were treated using parametric tests and expressed as mean and standard deviation.

In the *in vitro* experiment, two-way ANOVA was used, with Bonferroni *post hoc* when necessary (significant *F* ratios), to analyze possible statistical differences in the results related to food consumption, body mass, physical capacity tests, macrophage infection rates, and cytokine concentrations between different experimental groups (SED, LV, MV, HV, VHV, and TAP), moments (time of intervention), or conditions (with or without LPS). The paired *t*-test was used to compare the time to exhaustion in minutes between the VHV group (physical capacity test 3) and the TAP group (physical capacity test 4).

The two-way ANOVA for results related to food consumption (amount of feed in grams) and body mass (in grams) consisted of a 5 × 10 comparison (five experimental groups × 10 intervention weeks). For physical capacity tests (time to exhaustion in minutes), the two-way ANOVA consisted of a 5 × 3 comparison [five experimental groups × three moments (pre-training, during, and post-training)].

For macrophage infection factors and cytokine concentrations (pg/ml), the two-way ANOVA consisted of a 6 × 2 comparison [six experimental groups × two conditions (with or without LPS)].

The results obtained in the *in vivo* experimentation, except for the development of the injury, were analyzed by one-way ANOVA followed by Tukey’s multiple comparison test. Also analyzed by one-way ANOVA, followed by Tukey’s multiple comparison test, were the infection factor experiment obtained after 12 weeks of animal training and the results of the nitric oxide dosage resulting from this interaction.

The two-way ANOVA for the leishmaniotic injury development assay consisted of a 3 × 12 comparison (three experimental groups × 12 weeks of training).

All statistical procedures were performed using the GraphPad Prism 5.01^®^ software, assuming a significance level of *P* < 0.05.

## Results

### Food Consumption, Body Mass Assessment, and Physical Capacity Test

**FIGURE 1:**
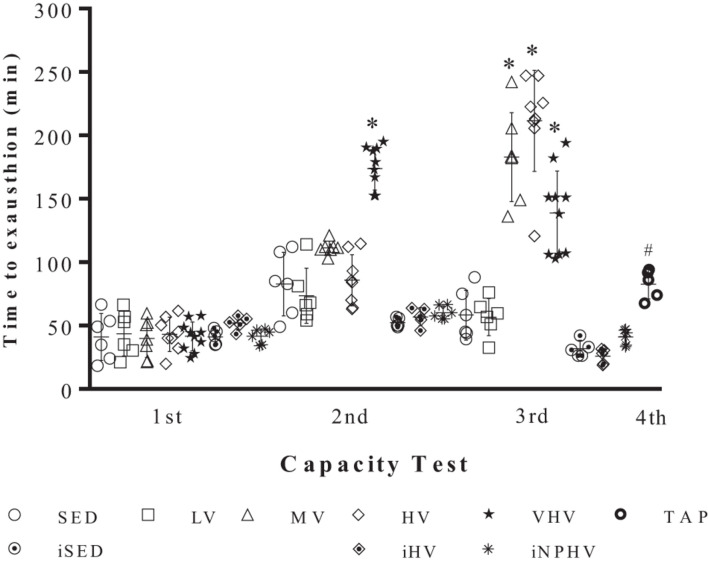
Physical capacity tests (time to exhaustion in minutes) between different experimental groups before (1st), during (2nd), and after (3th) the 10-week training period and 2 weeks of active rest. Values expressed as mean ± SE (^∗^*P* < 0.001). Groups: sedentary control (SED), low volume (LV), medium volume (MV), high volume (HV), very high volume (VHV), tapering (TAP), infect sedentary control (iSED), infected high volume (iHV), and infected non-periodized high volume (iNPHV).

There were no significant differences in body mass between the groups (sedentary and exercised) at any point from the initial to the final week. Even the increase in body mass due to the natural growth of mice was not significantly different between the initial and final weeks (Table 2). In the same way, there was no difference in the food consumption during all training time. There are no significant differences at time to reach exhaustion of the first physical capacity test. In the second test, the VHV group showed a significant difference from the other groups, showing better accomplishment in animal performance. During the third physical capacity test, the MV, HV, and VHV groups showed an increase in the time to reach exhaustion, showing a possible effect of physical exercise on the adaptation of these animals. The TAP group that comes from the VHV group showed a decrease in performance in their physical capacity test (Figure 1).

**TABLE 2:** Body mass in mice subjected to regulated training for 10 weeks (SED, LV, MV, HV, and HVH) and 12 weeks (TAP, iSED, iVH, and iNPHV).

	**SED**	**LV**	**MV**	**HV**	**VHV**	**TAP**	**iSED**	**iVH**	**iNPHV**
Initial week	30.15 ± 1.33	28.27 ± 1.98	26.32 ± 1.48	28.28 ± 1.47	27.3 ± 1.33	27.3 ± 1.33	27.35 ± 1.79	26.90 ± 1.93	27.90 ± 1.89
Final week	32.28 ± 1.81	32.12 ± 2.13	30.23 ± 1.11	32.78 ± 1.77	31.35 ± 1.81	32.58 ± 1.44	28.83 ± 1.34	27.17 ± 1.38	29.68 ± 1.97

*In vitro infection groups: sedentary control (SED), low volume (LV), medium volume (MV), high volume (HV), very high volume (VHV), and tapering (TAP).*

*In vivo infection groups: sedentary control (iSED), high volume (iHV), and non-periodized high volume (iNPHV).*

### Parasite–Macrophage Interaction (*in vitro*)

**FIGURE 2:**
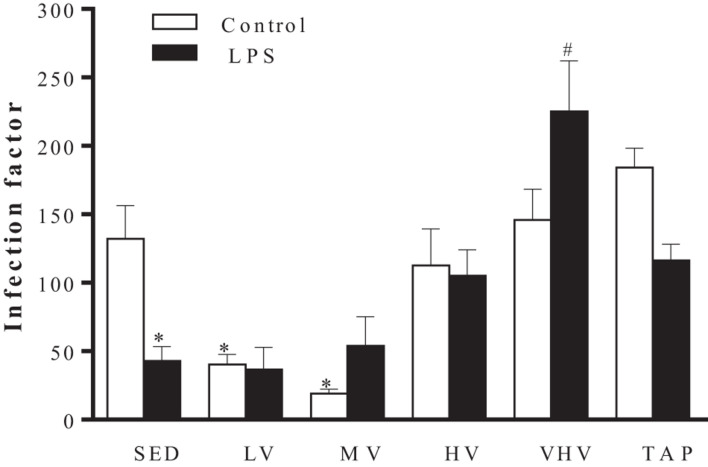
Effect of chronic physical exercise on the interaction between BALB/c mice peritoneal macrophages and *Leishmania major* promastigotes. Values represent the mean and standard error of at least five animals per group. ^∗^*P* < 0.001 in relation to the sedentary group without LPS and #*P* < 0.05 in relation to the sedentary group with LPS. Groups: sedentary control (SED), low volume (LV), medium volume (MV), high volume (HV), very high volume (VHV), and tapering (TAP).

Macrophages from animals submitted to 10 weeks of LV and MV exercises showed a significant decrease in the infection factor by *L. major*, 40.4 and 19, respectively, indicating a possible improvement in their leishmanicidal mechanisms (Figure 2). In the presence of LPS, macrophages from sedentary animals showed a significant decrease in the infection factor (from 132.2 to 42.8), while those submitted to the VHV exercise had the highest infection factor (225), which may be an indication of inhibition of its microbicide mechanisms (Figure 2). However, although there was a change in the infection factor of the groups, there was no change in the production of NO by macrophages in these animals (data not shown).

### Effect of Physical Exercise on Cytokine Production by Macrophages (*in vitro*)

**FIGURE 3:**
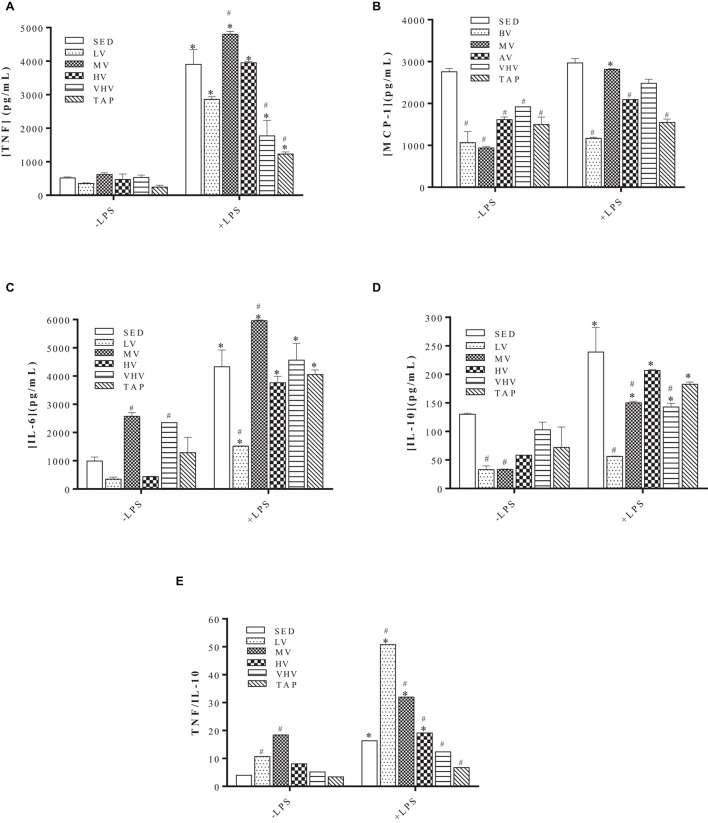
Effect of physical training on cytokine production by *Leishmania major*-infected macrophages. Macrophages were extracted from the mice peritoneum, plated in the absence or presence of bacterial lipopolysaccharide (LPS) 1 μg/ml for a period of 24 h, and then infected with *L. major* (five parasites:one macrophage), for an equal period of 24 h. Values represent the mean and standard error of two experiments with at least six animals per group. ^∗^*P* < 0.05 relative to their respective untreated control with LPS. #*P* < 0.05 in relation to their respective SED controls. Groups: sedentary control (SED), low volume (LV), medium volume (MV), high volume (HV), very high volume (VHV), and tapering (TAP). **(A)** TNF, **(B)** MCP-1, **(C)** IL-6, **(D)** IL-10, and **(E)** TNF/IL-10 ratio.

There was no change in TNF production by infected macrophages non-treated with LPS; however, all groups showed an increase in TNF production after LPS treatment. Nevertheless, MV exercise promoted an increase in the production of this cytokine when macrophages from this group were compared with the SED + LPS group. On the other hand, macrophages from the groups submitted to VHV and TAP exercise presented a decrease in the production of TNF, in comparison to the SED + LPS group (Figure 3A).

Infected macrophages from trained animals produced less MCP-1 compared with the SED group. When macrophages were pretreated with LPS, we observed that only the MV group produced MCP-1 levels similar to those of the SED group, while the other groups produced less (Figure 3B).

Macrophages not activated by LPS and originating from groups MV and VHV had their production of IL-6 increased in comparison with the SED group. However, only MV showed an increase in the production of this cytokine after LPS treatment (Figure 3C), where the LV group showed a decrease in IL-6 compared with the SED + LPS group (Figure 3C).

Macrophages of trained groups LV and MV, without LPS pretreatment, produced significantly less IL-10 when compared with the SED group. When pretreated with LPS, macrophages of the LV, MV, and TAP groups had their IL-10 production decreased (Figure 3D).

The TNF/IL-10 ratio showed that the LV and MV groups, pretreated or not with LPS, presented values significantly higher than those obtained by the sedentary (SED) group and the other groups submitted to higher volumes of exercise (Figure 3E), strongly suggesting a predominance of the Th1-type response in these groups. The groups submitted to VHV and TAP exercise presented a lower ratio than that found in the SED group (Figure 3E), suggesting that the Th1/Th2 balance in these groups may be leaning more toward the production of cytokines more similar to those found in the Th2 pattern.

### Effect of High Volume and Non-periodized High Volume Training on the Development of Experimental *L. major* Infection in BALB/c Mice (*in vivo*)

**FIGURE 4:**
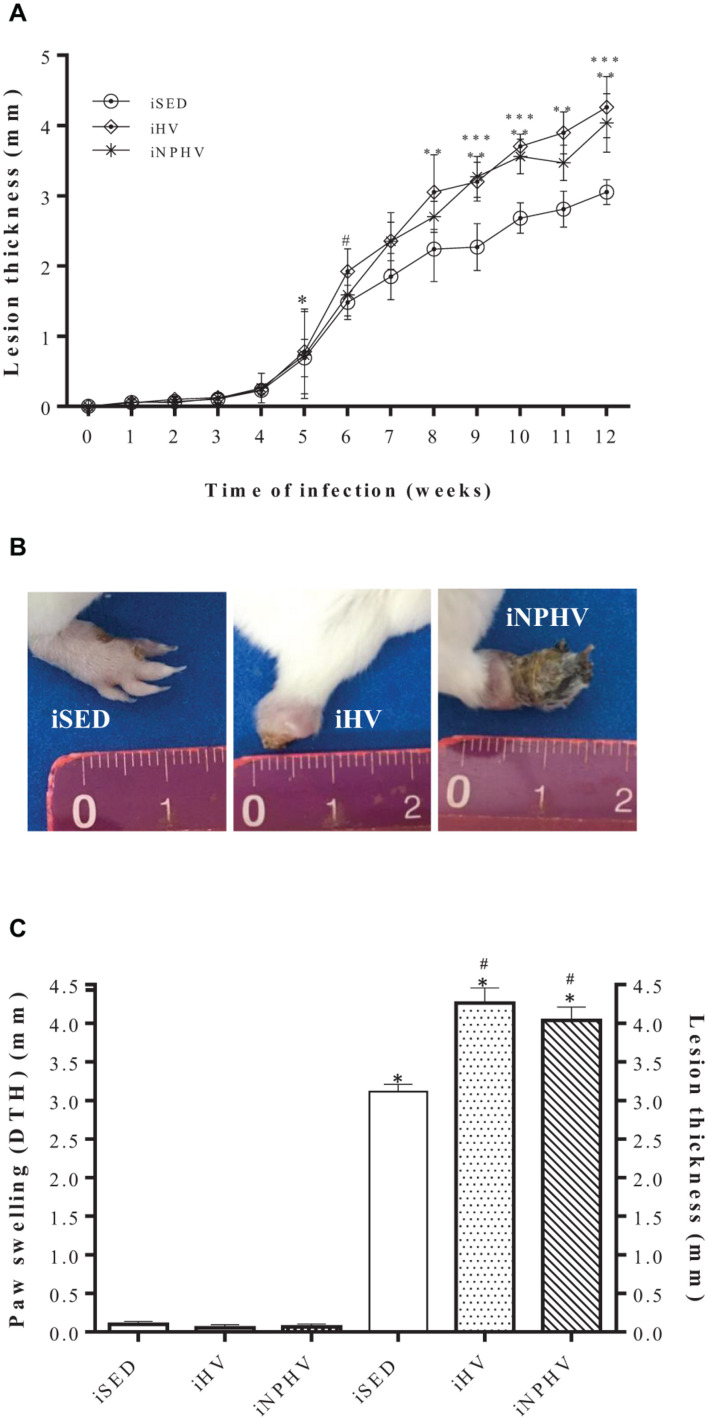
Effect of physical exercise on the development of the paw lesion in a BALB/c mouse infected with *L. major* (2 × 10^6^ promastigotes). **(A)** The paws were measured weekly with the aid of a caliper (Mitutoyo). The expressed results represent the difference between the measure of the contralateral paw (mm) and the infected paw, during 12 weeks of training with *in vivo* infection. Values represent the mean of the animals in each group and the bars represent the standard error. The ^∗^ indicates from which week the lesion became significant for iHV and iNPHV (5th week) and the # indicates from which week the lesion became significant for iSED (6th week). ^∗∗^ iHV with *P* < 0.0001 in relation to sedentary control (iSED) from the 8th week. ^∗∗∗^ iNPHV with *P* < 0.0001 compared with sedentary control (iSED) in weeks 9, 10, and 12. **(B)** Photographs are representative of one animal from each group. Photographs taken in the 13th week. **(C)** Comparison between DTH (48 h) and lesion thickness in paws after 12 weeks of *L. major* infection. ^∗^*P* < 0.0001 in relation to the respective DTH values. #*P* < 0.0001 in relation to the size of the sedentary control lesion. Groups: infected sedentary control (iSED), infected high volume (iHV), and infected non-periodized high volume (iNPHV).

The HV and NPHV exercises promoted an increase in the development of the leishmaniotic lesion; in addition, in trained animals (iHV and iNPHV), the onset of the lesion occurred 1 week before that in sedentary animals (iSED). That is, in the animals of the iHV and iNPHV groups, the thickness of the infected paw became significantly different from the thickness of the still uninfected paw (0 week of infection) in the 5th week post-infection, while in the animals of the sedentary control group (iSED), this difference only became significant after the 6th week post-infection (Figure 4A). The HV exercise promoted a significant increase in the thickness of the injured paw from the 8th week, while the NPHV exercise promoted this effect from the 9th week (Figure 4A).

In addition to the increase in the lesion thickness, the physical exercise of HV and NPHV provided an increase in necrosis in the infected paws, even promoting loss of necrotic parts in the paw of some animals, as in this example of the animal in the iHV group (Figure 4B). Despite appearing to trigger an exacerbated inflammatory process, physical exercise did not promote a delayed hypersensitivity response in animals, as shown by the DTH test carried out (Figure 4C).

### Parasite Load and Visceralization of the Infection

**FIGURE 5:**
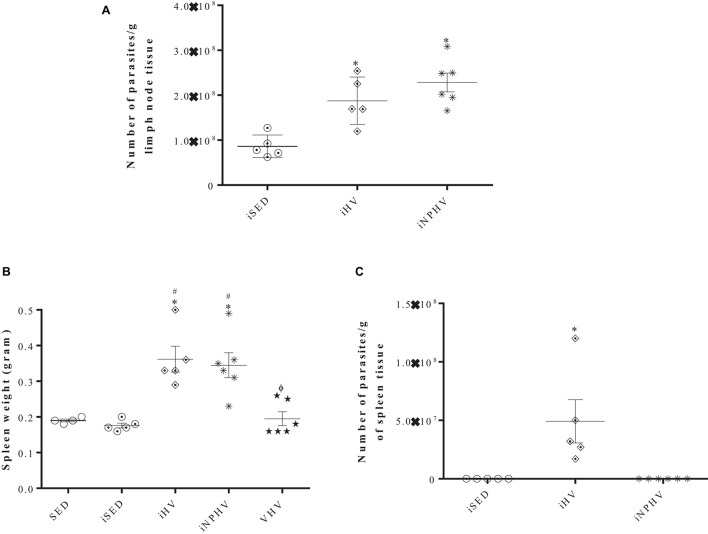
Effect of physical training on the parasite load of lymph nodes **(A)** and on the visceralization of leishmaniasis by *L. major* in BALB/c mice (presence of parasites in the spleen of infected mice) **(B,C)**. The parasites were isolated from lymph nodes and spleens after 12 weeks of infection and quantified by limiting dilution in Schneider’s medium plus 20% FBS. **(A)** After isolation, there was growth of the sedentary control system (iSED) in the last dilution after 24 h of incubation, when all groups were quantified and the number of parasites was divided by the lymph node weight (in grams). **(B)** Weight of spleens. **(C)** After isolation, there was growth of promastigotes in the iHV group after 4 days of incubation; the parasites were quantified by counting in a Neubauer chamber and its number was divided by the weight (in grams) of the spleen. The plates were kept at 26°C during 14 days to verify the parasite growth in the iSED and iNPHV groups. Values represent mean and bars represent standard error. In panel **(A)**, ^∗^*P* < 0.0006 in relation to iSED. In panel **(B)**, ^∗^*P* < 0.0001 in relation to SED; #*P* < 0.0001 in relation to iSED. Φ*P* < 0.0001 in relation to groups iHV and iNPHV. In panel **(C)**, ^∗^*P* < 0.0057 in relation to sedentary control (iSED) and iNPHV. Groups: sedentary control (SED), very high volume (VHV), infected sedentary control (iSED), infected high volume (iHV), and infected non-periodized high volume (iNPHV).

The animals submitted to the HV (iHV) and NPHV (iNPHV) exercise showed a significant increase in the parasite load of the lymph nodes draining the lesion, going from about 1 × 10^8^ to approximately 2 × 10^8^ and 2.4 × 10^8^ parasites/g of tissue, respectively (Figure 5A), although there were no changes in lymph node size (data not shown).

After the euthanasia of the animals from the SED, iSED, iHV, iNPHV, and VHV groups, their spleens were obtained, weighted, and macerated, and the resulting material was incubated at 26°C to verify the infection visceralization. The spleens of the animals from the iHV and iNPHV groups were heavier than the others, weighing about 0.362 and 0.345 g, respectively, while the spleens of the other groups weighed about 0.19 g (Figure 5B). After 4 days of incubation, we observed parasite growth just in the spleen cell medium (approximately 5 × 10^7^ parasites/g of spleen tissue) from the iHV animals (Figure 5C).

### Effect of Physical Exercise of HV and NPHV on Cytokine Production by Spleen Lymphocytes

**FIGURE 6:**
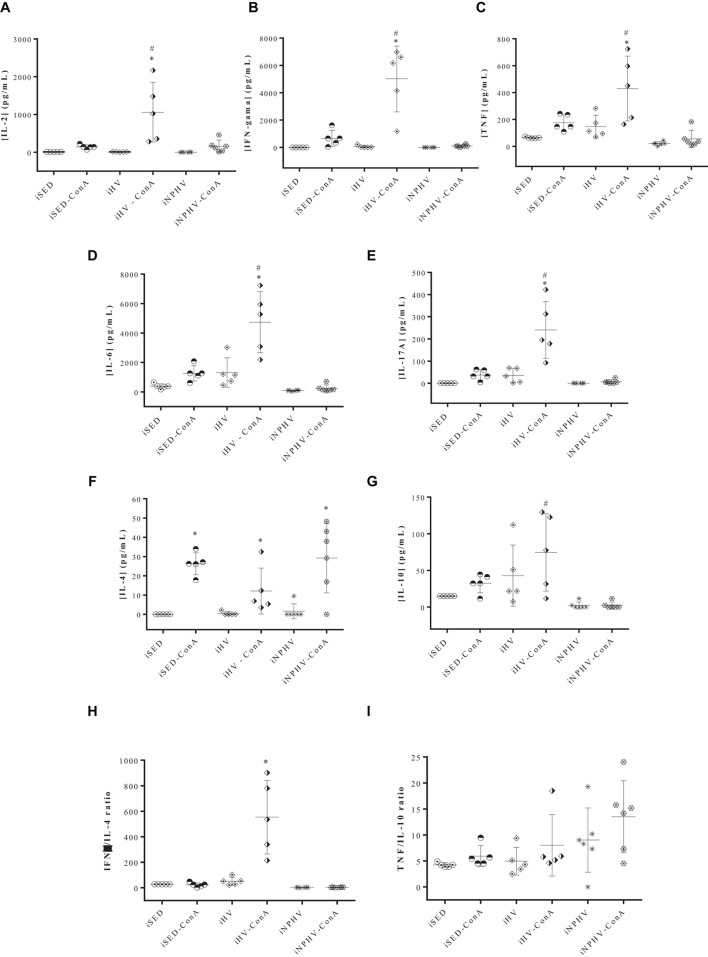
Effect of physical exercise on cytokine production by spleen cells of BALB/c mice. The concentration of cytokines was determined by the CBA method, according to the section “Materials and Methods.” Values represent the mean of two experiments with samples of at least five animals per group, and the bars represent the standard error. **(A)** IL-2; **(B)** IFN-γ; **(C)** TNF; **(D)** IL-6; **(E)** IL-17A; **(F)** IL-4; **(G)** IL-10; **(H)** IFN-g/IL-4 ratio; **(I)** TNF/IL-10 ratio. ^∗^*P* < 0.005 in relation to its own control without ConA; #*P* < 0.005 in relation to its iSED group. Groups: infected sedentary control (iSED), infected high volume (iHV), and infected non-periodized high volume (iNPHV). ConA, concanavalin A.

Spleen cells stimulated by mitogen concanavalin A (ConA) significantly increased the production of almost all cytokines (IL-2, IFN-γ, TNF, IL-6, IL-17A, and IL-4) measured from animals submitted to HV exercise (Figures 6A–F), with the exception of IL-10 (Figure 6G). The HV exercise stimulated the production of IL-2, IFN-γ, TNF, IL-6, IL-17A, and IL-10, while the NPHV exercise practically inhibited the production of all cytokines.

The IFN-γ/IL-4 ratio showed that spleen lymphocyte stimulated by ConA of animals submitted to HV exercise presented a cytokine production type closer to the Th1 pattern (Figure 6H). The TNF/IL-10 ratio, on the other hand, showed no significant differences between the groups (Figure 6I).

## Discussion

Physical exercise has been associated with numerous biological and functional benefits (Fiuza-Luces et al., 2013). There are potentially beneficial intense protocols, such as high-intensity interval training (Burgomaster et al., 2005, 2008; Gibala and McGee, 2008). However, the desirable effects of exercise seem to depend on an adequate dosage, that is, a dose capable of not causing harmful side effects. Short recovery periods between exercise sessions, in addition to increased training volume and intensity, mental stress, and poor diet, are related to impairment not only of athletic performance but also of health (Cadegiani and Kater, 2017, 2019).

The use of extreme loads can cause negative reactions in the exercise adaptation process (Gomes, 2009). The adaptive process is not a linear function of the physical load, and there is an individual adaptation limit (Gomes, 2009). The decrease in performance as a result of the use of extreme loads can be the main outcome, being considered a consensual factor for finding overtraining (Meeusen et al., 2013).

Since the 1990s, the “women athlete triad” has been debated by the American College of Sports Medicine (Otis et al., 1999; Nattiv et al., 2007). This syndrome is defined by the presence of anorexia, amenorrhea, and osteoporosis, in general, resulting from the overtraining syndrome, and considered a neuroendocrine disorder (Otis et al., 1999; Nattiv et al., 2007). The increased production of endogenous opioids and beta-endorphin promoted by physical activity can stimulate a decrease in the production of gonadotropin-releasing hormone (GnRH) and a consequent decrease in the release of gonadotropins (FSH and LH), which determines the drop in estrogen levels (Nattiv et al., 2007), which in addition to menstrual alterations can interfere with the immune response, as estrogen regulates immune response *via* modulation of endosomal TLRs and TLR8 expression; thus, hormonal balance determines the overall response in females (Marriott et al., 2006; Roberts et al., 2012; García-Gómez et al., 2013; Young et al., 2014). Although the concept “relative energy deficiency in sport” (RED-S) is considered more appropriate than “women athlete triad,” as it reflects something of greater complexity, which affects physiological, psychological, physical, and mental health aspects, in addition to performance itself, between people of different ages and genders (Mountjoy et al., 2014), we decided to carry out the experiments using only male mice to avoid possible interferences arising from hormonal variation presented by females.

The group submitted to VHV exercise presented the best performance in the second test; however, at the end of 10 weeks of training, its performance got worse (Figure 1). To verify if the animals were in overtraining or overreaching, they were submitted to a tapering period of 2 weeks with a significant reduction of exercise volume. The overreaching can be understood as a temporary exhaustion promoted by the excessive physical training and stress, which results in the partial diminishing in the performance. Nevertheless, the recuperation occurs within 2–4 weeks (Halson and Jeukendrup, 2004).

Intensified training is often used to improve performance. When there is an appropriate recovery, an overcompensation effect of physiological adaptations may occur, followed by an improvement in performance compared with baseline levels (Meeusen et al., 2013). We verified that the TAP group had even worse performance in relation to the VHV group, suggesting the occurrence of overtraining and the confirmation that the type of training adopted in this group was strenuous.

The “unexplained underperformance syndrome,” a term proposed for the replacement of overtraining syndrome, was defined as a persistent decrease in performance capacity despite a period (2–4 weeks) of relative rest (Meeusen et al., 2013). This condition can be caused by the excessive release of cytokines during and after exercise (“cytokine disease”), causing a chronic inflammatory state (Robson, 2003). Gholamnezhad et al. (2014) found that 2 weeks of gradual reduction in training loads was not enough to reverse the cellular immunosuppression caused by the targeting of the cytokine profile to a humoral response in rats (Gholamnezhad et al., 2014).

The animals submitted to MV physical exercise showed a lower rate of infection even in the absence of LPS stimulus, a known macrophage activator. The LV group also showed a decrease in this infection factor (Figure 2). On the other hand, VHV training promoted a significant increase in the infection factor, even in macrophages activated by LPS (Figure 2). This effect could perhaps be explained, at least partially, by the increased production of IL-6 in macrophages promoted by this volume of exercise (Figure 3C). In animal testing, IL-6 promotes Th2 responses in CL (Moskowitz et al., 1997; Saha et al., 1999; Maspi et al., 2016). In this case, this cytokine could inhibit the microbicidal action of the macrophage.

The sedentary group (SED) had a higher infection factor than those presented by mice that were submitted to the LV and MV exercise and lower than those submitted to the VHV exercise and the TAP group (Figure 2).

Macrophages from the group submitted to MV exercise showed a greater production of inflammatory cytokines (Figures 3A,C), except for the chemokine MCP-1 (Figure 3B). Its production of IL-10, a known anti-inflammatory cytokine, was significantly decreased as a result of MV exercise (Figure 3D). These data point to a predominance of a Th1-type response over a Th2 one, as can be seen in the graph of the TNF/IL-10 ratio (Figure 3E). This is also the case for the LV group, whose TNF/IL-10 ratio was the highest in LPS-activated macrophages (Figure 3E). On the other hand, macrophages from the groups of mice submitted to VHV exercise and to the exercise volume reduction process (TAP) demonstrate a greater predominance of the Th2 cytokine profile over the Th1 profile (Figures 3A,B,E). The increase in Th2 cytokine profile must be correlated with the increase in macrophage infection observed in the VHV and TAP groups. These data are in agreement with previous studies where moderate-intensity exercise promoted a predominance of Th1 cytokine production in ConA-stimulated lymphocyte supernatants, as well as in the LPS-activated macrophage supernatant of trained BALB/c mice for 12 weeks (Terra et al., 2012a).

The balance between the Th1 and Th2 standard cytokines is important because IL-10 can impair the production of IL-12 by macrophages, inhibiting the microbicidal functions of this phagocyte (Bashyam, 2007). On the other hand, this balance can contribute to a protective immune response, in case of balance between inflammatory and anti-inflammatory cytokines, or promote a deleterious effect, with tissue destruction when there is an exacerbation of the immune response, in case of hyperergy (Gomes-Silva et al., 2007). The findings of the present study are in agreement with the literature, as the moderate combination of exercise frequency, duration, and intensity promoted an improvement in macrophage function.

In the study by Ceddia and Woods (1999), it was found that mice experienced a suppression of antigen presentation by macrophages after 4 days of exhaustive training (Ceddia and Woods, 1999). Studies with a longer intervention time, such as the one by Ru and Peijie (2009), where rats were submitted to 9 weeks of progressive training, also verified a cellular immunosuppression with a predominance of humoral profile (Ru and Peijie, 2009). The anti-inflammatory effect prevents tissue damage caused by inflammatory mediators and reduces the risk of chronic inflammatory diseases, but may increase the susceptibility to infections by intracellular microorganisms (Elenkov, 2004; Gleeson, 2006; Terra et al., 2012a). On the other hand, moderate training can balance the pro- and anti-inflammatory responses, with a slight prevalence of the cellular profile over the humoral one, favoring the control of infections caused by intracellular microorganisms (Gleeson, 2006; Terra et al., 2012a,b).

In the same way, 8 weeks of moderate training and 11 weeks of severe training (overtraining) altered the plasma cytokine concentrations of uninfected rats. Moderate training promoted the production of cytokines linked to cellular immunity, while in the severe training group and in sedentary control, a response toward the Th2 profile was observed (Gholamnezhad et al., 2014).

The HV and NPHV exercises promoted an increase in the susceptibility of BALB/c mice to *L. major* infection (Figures 4A,B). Previous works show that high-intensity exercise can elicit a Th2 anti-inflammatory response and favor infections by intracellular microorganisms (Elenkov, 2004; Gleeson, 2006). BALB/c mice naturally exhibit a Th2-type cytokine pattern (Dutra and Da-Silva, 2017) and exercise seemed to further stimulate this pattern (Figures 3, 4). In previous results, 12 weeks of moderate-intensity exercise inhibited the development of infection in BALB/c mice by parasites of the *L. major* species, reversing the pattern of predominance from Th2 to Th1 in BALB/c mice (Terra et al., 2019).

When we performed the delayed hypersensitivity (DTH) test, none of the infected animals showed a positive response (Figure 4C). Although it is not a definitive proof, non-responsiveness to *Leishmania* antigens strongly suggests that there was no cellular immune response. On the contrary, BALB/c mice infected with *L. major* and submitted to 12 weeks of moderate-intensity exercise presented a positive DTH response to the antigens of this parasite (Terra et al., 2019).

The paws of the animals were severely injured, showing tissue destruction and loss of the paw tissue due to the great inflammatory response presented by animals submitted to the exercise of HV and NPHV. In this way, unfortunately, we were unable to quantify the parasite load on the injured paws. However, we quantified the parasite load in the draining lymph nodes of these lesions. It is known that even in cured animals, the persistence of parasites in the draining lymph node occurs for long periods. This persistence seems to be important for the maintenance of the cellular immune response and T-cell memory generation (Mandell and Beverley, 2016; Conceição-Silva et al., 2018). The HV and NPHV exercises promoted a significant increase in the number of parasites/g of lymphoid tissue (Figure 5A). On the other hand, moderate-intensity exercise was able to strongly reduce the number of parasites isolated from the paw of infected animals (Terra et al., 2019), corroborating, once again, the hypothesis that moderate exercise promotes the predominance of Th1 and that exercises of greater intensity or volume promote a predominance of a Th2 response.

When analyzing the weight of the spleens of animals in the sedentary groups (SED or iSED), iHV, iNPHV, and VHV, we observed that exercise increased the size of the spleens only in the infected animals (Figure 5B), suggesting a greater proliferation of lymphoid tissue and the possible visceralization of the infection. However, the parasites only grew in cultures of splenic cells isolated from the spleens of animals in the group submitted to HV exercise (iHV), confirming our hypothesis of visceralization only in this group. In the culture of cells from the spleen of animals from the infected sedentary group and from the iNPHV group, there was no growth of parasites. Thus, visceralization occurred only in animals submitted to training with HV exercise (Figure 5C). The possibility of metastasis and visceralization of infection in BALB/c mice by *L. major* has been described in the literature since the late 1970s (Dutra and Da-Silva, 2017), and the performance of HV exercise facilitated this process.

The exercise of HV promoted an increase in the production of all measured cytokines (IL-2, IFN-γ, TNF, IL-6, IL-17A, and IL-10) by spleen cells, with the exception of IL-4 (Figure 6G), whose values had no significant difference between the three different groups. The TNF/IL-10 ratio (Figure 6I) also showed no significant difference between the exercised and sedentary groups, possibly showing a greater balance between the cross-reactions of cellular immune responses of the Th1 and Th2 types. However, the IFN-γ/IL-4 ratio highlighted the animals in the HV group as showing a predominance of the Th1 pattern (Figure 6H). A result similar to the latter was obtained when BALB/c mice, submitted to 12 weeks of moderate-intensity exercise, presented IFN-γ/IL-4 and IFN-γ/IL-10 ratios clearly biased toward a Th1 response in lymph node cells. These data suggest that moderate-intensity exercise is able to modulate the Th1 response that provides a protective effect against the development of lesions promoted by *L. major* (Terra et al., 2019), while an exacerbated predominance of the production of inflammatory cytokines, in the case of animals submitted to HV exercises, can contribute to tissue destruction observed in the paws of infected BALB/c mice of the iHV group, rather than protecting them from infection and tissue damage caused by *L. major*, since IFN-γ production in this group (Figure 6B), for example, was about 45 times greater than that found in animals submitted to moderate exercise (Terra et al., 2019). As seen in this work, lymphocytes from uninfected animals trained for 12 weeks show a type 1 cytokine profile after mitogenic stimulation (Terra et al., 2012a).

The cytokine IL-6 is mainly produced by monocytes, macrophages, and muscle cells. It can be considered pro- and anti-inflammatory. Some of its effects include respiratory burst in neutrophils, acute-phase protein production, inhibition of IL-1 and TNF, glucose uptake in skeletal muscle, lipolysis in muscle and adipose tissue, and hepatic gluconeogenesis (Terra et al., 2012b). Our findings indicated that the two groups with the highest production of IL-6 by macrophages were HV and VHV, and by lymphocytes, the HV group, confirming the important regulatory role of this cytokine for the preservation of balance or its restoration in the face of a severe disturbance of homeostasis.

Although the volume of exercise applied to the animals in the iNPHV group was a little bit greater than in the iHV group, the iNPHV group did not show an increase in the production of cytokines, presenting very low levels of them. Possibly, one of the explanations for this phenomenon could be the exhaustion of T lymphocytes, a process already observed in cases of infections by parasites of the genus *Leishmania* (Egui et al., 2018; Valadares et al., 2018; reviewed by Pérez-Antón et al., 2019), including the species *L. major* (Valadares et al., 2018). Exhaustion of immune system cells can occur due to antigenic hyperstimulation, affecting their activation, transit, and cell functions (Santana et al., 2008; Yi et al., 2010; Silva et al., 2012; Lima et al., 2014). Several lines of evidence show the presence of positive cell depletion markers, such as inhibitory receptors, during chronic *Leishmania* infection. This increased expression can be observed in both CD4^+^ and CD8^+^ T lymphocytes. In addition to the presence of these markers, the loss of the specific functional capacity of these lymphocytes is also verified, and a positive correlation between this cell exhaustion and the severity of the parasitosis can be observed (reviewed by Pérez-Antón et al., 2019). Possibly, the increase in the parasite load observed in the iNPHV group (Figure 5A) may have promoted antigenic hyperstimulation, leading to lymphocyte exhaustion. However, there was no significant difference between the parasite load observed in lymph nodes from the iHV and iNPHV groups (Figure 5A). Furthermore, the visceralization of the infection occurred only in the group submitted to the HV exercise (iHV). Thus, depending on the exposure to antigens, it might be more plausible to expect the exhaustion of immune system cells in this group (iHV).

There are several works in the literature relating the susceptibility or resistance to infection by parasites of the *Leishmania* genus with the cellular immune response (Mosmann et al., 1986; Barthelmann et al., 2012; Terra et al., 2012a, 2019). Generally, individuals with a balanced cellular immune response have a good prognosis in the case of American tegumentary leishmaniasis (Brasil, 2013). Susceptibility, on the other hand, has been associated with a non-protective Th2-type (T helper 2) lymphocyte activation response, with production of IL-4 and IL-10 (Bogdan et al., 1996; Launois et al., 1997; Romagnani, 1997; Mansueto et al., 2007). However, in case of hyperergy of the cellular immune response (Th1), there is a poor prognosis, with exacerbated destruction of the host tissue, where the parasitic antigens are found, a very characteristic situation in cases of mucosal leishmaniasis. In this clinical manifestation, the concentrations of IFN-γ and TNF are very high and there is a relatively low production of IL-10 and IL-4. Furthermore, cells infected by parasites of the *Leishmania* genus generally have low responsiveness to cytokines that inhibit IFN-γ secretion (Brasil, 2013). Possibly, the large tissue destruction observed in the paws of animals from the iHV group (Figure 4B) is due to the high production of inflammatory cytokines with a relatively low production of IL-4 and IL-10 (Figure 6). It is also possible that a high concentration of pro-inflammatory cytokines was produced at the site of infection, contributing to tissue damage.

Few studies have tested the hypothesis that the immune system is chronically altered by excessive stress caused by exercise. Excessive chronic stress caused by physical training can generate cellular immunosuppression and increase the susceptibility to infections, which can compromise health as well as a sedentary lifestyle. Physical exercise has been considered the “best medicine” (Deweerdt, 2011) due to epidemiological evidence related to public health. However, its effectiveness is frequently questioned in the experimental area. Regular exercise has benefits until the development of chronic exhaustion occurs, whose operational definition differs among authors and whose physiological process remains poorly understood to this day (Meeusen et al., 2006, 2013; Farhangimaleki et al., 2009; Cadegiani and Kater, 2019). The paradigm that only elite athletes experience symptoms of the overtraining syndrome seems to prevail. This condition is similar to some non-communicable chronic diseases and has a multifactorial etiology, wide variation between individuals, and non-specificity, and its development is not restricted to classic training variables (frequency, duration, intensity, and interval of sessions) (Meeusen et al., 2013; Guimarães et al., 2017, 2018; Cadegiani and Kater, 2019). The idea that “the more the better” may be an exercise adherence strategy incompatible with the balance necessary for the full functioning of organic systems.

High volume, very high volume, and a non-periodized high volume aerobic training compromised long-term physical capacity. Both these types of training and physical inactivity were conditions that increased the susceptibility of *in vitro* macrophage infections and during an *in vivo* experiment. Coherent load distribution through moderate aerobic training was the best strategy for promoting cellular immune response and improving physical capacity. Physical exercise can have paradoxical effects and its consistent prescription depends on a better understanding of its cellular mechanisms.

## Data Availability Statement

The original contributions presented in the study are included in the article/supplementary material, further inquiries can be directed to the corresponding author.

## Ethics Statement

The animal study was reviewed and approved by Ethics Committee on Experimental Use and Animal Care of the Instituto de Biologia Roberto Alcântara Gomes - UERJ (CEUA 019/2015).

## Author Contributions

TG conducted the experiments and contributed to the writing of the manuscript. SG, RA, AL, JS, MA, AB, RS, TS, LS, BV, and RT conducted the experiments. LR contributed to the execution and discussion of cytokine production analysis. SS conducted the experiments and co-orientation and contributed to the writing of the manuscript. PD conducted the orientation, experimentation, and writing of the manuscript. All authors contributed to the article and approved the submitted version.

## Conflict of Interest

The authors declare that the research was conducted in the absence of any commercial or financial relationships that could be construed as a potential conflict of interest.

## Publisher’s Note

All claims expressed in this article are solely those of the authors and do not necessarily represent those of their affiliated organizations, or those of the publisher, the editors and the reviewers. Any product that may be evaluated in this article, or claim that may be made by its manufacturer, is not guaranteed or endorsed by the publisher.
